# Anti-VEGF Drugs Influence Epigenetic Regulation and AMD-Specific Molecular Markers in ARPE-19 Cells

**DOI:** 10.3390/cells10040878

**Published:** 2021-04-12

**Authors:** Mohamed A. Hamid, M. Tarek Moustafa, Sonali Nashine, Rodrigo Donato Costa, Kevin Schneider, Shari R. Atilano, Baruch D. Kuppermann, M. Cristina Kenney

**Affiliations:** 1 Gavin Herbert Eye Institute, University of California Irvine, Irvine, CA 92697, USA; drmohamedhamid83@mu.edu.eg (M.A.H.); mohamedtarek@mu.edu.eg (M.T.M.); snashine@uci.edu (S.N.); rodrigodonato@yahoo.com.br (R.D.C.); kschneid@hs.uci.edu (K.S.); satilano@hs.uci.edu (S.R.A.); bdkupper@uci.edu (B.D.K.); 2 Ophthalmology Department, Faculty of Medicine, Minia University, Minia 61111, Egypt; 3 Instituto Donato Oftalmologia, Poςos de Caldas, MG 37701-528, Brazil; 4 Department of Biomedical Engineering, University of California Irvine, Irvine, CA 92697, USA; 5 Department of Pathology and Laboratory Medicine, University of California Irvine, Irvine, CA 92697, USA

**Keywords:** AMD, age-related macular degeneration, trichostatin A (TSA), HDAC, histone deacetylase, vascular endothelial growth factor (VEGF)

## Abstract

Our study assesses the effects of anti-VEGF (Vascular Endothelial Growth Factor) drugs and Trichostatin A (TSA), an inhibitor of histone deacetylase (HDAC) activity, on cultured ARPE-19 (Adult Retinal Pigment Epithelial-19) cells that are immortalized human retinal pigment epithelial cells. ARPE-19 cells were treated with the following anti-VEGF drugs: aflibercept, ranibizumab, or bevacizumab at 1× and 2× concentrations of the clinical intravitreal dose (12.5 μL/mL and 25 μL/mL, respectively) and analyzed for transcription profiles of genes associated with the pathogenesis age-related macular degeneration (AMD). HDAC activity was measured using the Fluorometric Histone Deacetylase assay. TSA downregulated *HIF-1α* and *IL-1β* genes, and upregulated *BCL2L13, CASPASE-9,* and *IL-18* genes. TSA alone or bevacizumab plus TSA showed a significant reduction of HDAC activity compared to untreated ARPE-19 cells. Bevacizumab alone did not significantly alter HDAC activity, but increased gene expression of *SOD2, BCL2L13, CASPASE-3,* and *IL-18* and caused downregulation of *HIF-1α* and *IL-18*. Combination of bevacizumab plus TSA increased gene expression of *SOD2, HIF-1α, GPX3A, BCL2L13,* and *CASPASE-3*, and reduced *CASPASE-9* and *IL-β*. In conclusion, we demonstrated that anti-VEGF drugs can: (1) alter expression of genes involved in oxidative stress (*GPX3A* and *SOD2*), inflammation (*IL-18* and *IL-1β*) and apoptosis (*BCL2L13, CASPASE-3,* and *CASPASE-9*), and (2) TSA-induced deacetylation altered transcription for angiogenesis (*HIF-1α*), apoptosis, and inflammation genes.

## 1. Introduction

Pathological angiogenesis, which subsequently leads to choroidal neovascularization, subretinal fibrosis, and exudative hemorrhage, is an underlying cause of the severe, late-stage, wet form of AMD (Age-related Macular Degeneration) [1].

Wet or neovascular AMD, which accounts for 10–20% of cases, is the less common of the two types of AMD. However, 90% of AMD-associated irreversible vision loss is attributed to wet AMD [2]. Dry AMD is characterized by degeneration of Retinal Pigment Epithelial (RPE) cells and accounts for 80% of AMD cases.

VEGF (Vascular Endothelial Growth Factor) is a signaling growth factor for vascular endothelial cells and a critical angiogenic factor that stimulates ocular neovascularization. Therefore, the most widely used wet AMD treatment targets the pro-angiogenic activity of VEGF to inhibit ocular neovascularization [3]. Administration of intravitreal injections of anti-VEGF drugs, such as ranibizumab (Lucentis), bevacizumab (Avastin), and aflibercept (Eylea), to wet AMD patients is successfully and routinely being used as a wet AMD therapy worldwide [4,5]. Despite the widespread use of anti-VEGF drugs, 10–15% of patients fail to respond to rigorous treatment protocols in clinical trial settings [6,7,8,9,10]. This variability in AMD patients’ response to therapy has been attributed to several clinical, behavioral, and genetic factors [11]. Pharmacogenetic studies have identified VEGFA, VEGFR2 (VEGF Receptor 2), CFH (Complement Factor H), and ARMS2 (Age-Related Maculopathy Susceptibility 2) as potential biomarkers for response to anti-VEGF drugs [12]. Many investigators, including the CATT (Comparison of Age-related Macular Degeneration Treatments) and IVAN (Inhibition of VEGF in Age-related choroidal Neovascularisation) research groups, did not find any significant association between genes polymorphism and visual or anatomic responses to treatment [13,14,15,16,17]. This inconsistency in findings by pharmacogenetic studies could be explained in part by possible gene–gene or gene–environmental interactions [18,19].

Genetic and environmental factors contribute to the development and progression of AMD. Genome-wide association studies (GWAS) have identified 52 genetic variants distributed across 34 loci associated with AMD [20]. Furthermore, epigenetic modifications, which include DNA methylation, histone acetylation/deacetylation, non-coding RNA-mediated gene silencing, and chromatin remodeling [21] have been implicated in the pathogenesis of AMD by selective transcription of genes involved in angiogenesis, inflammation, and oxidative stress pathways [22,23]. Epigenetic mechanisms result in covalent modifications in the DNA and regulate gene transcription either by activation or repression, in response to environmental stimuli and are often heritable [24]. Epigenetics can elucidate gene–environment interactions and explain why a certain genotype frequently results in different phenotypes [25]. Histone acetylation is catalyzed by histone transferases (HATs) and acts to destabilize nucleosomes and unwrap DNA to make it accessible to transcription factors. Conversely, histone deacetylation, carried out by histone deacetylases (HDACs), stabilizes nucleosomes and represses DNA transcription [26]. Histone acetylation is known to regulate the expression of 2–10% of genes. Other non-histone proteins, particularly transcription factors, are also regulated by acetylation/deacetylation. This could explain the fact that gene expression is not always silenced by deacetylation [27].

AMD being a leading cause of blindness in the United States and the third major cause of visual impairment worldwide, [28] poses a major health risk to the elderly population, and AMD risk is projected to increase by 54% in the United States in the next five year [29]. Therefore, we speculate that delving into the mechanisms of action of the currently used anti-VEGF drugs might contribute to the design of more effective therapeutic strategies for wet AMD. To this end, the current in vitro study was designed to examine the effects of anti-VEGF drugs on epigenetic regulation in immortalized human ARPE-19 cell lines. The ARPE-19 cell line used in this study was originally developed from the retinal pigment epithelium (RPE) of a human donor eye and resembles the phenotype and properties characteristic of aged native human RPE cells, lack of pigmentation, weak tight junctions, reduced expression of all-trans retinol, Pigment-Epithelium-Derived Factor (PEDF), and RPE markers, and hypersensitivity to VEGF activity, thereby making the ARPE-19 cell line an ideal in vitro AMD model [30]. However, it should be emphasized that the ARPE-19 cell line loses some of the aging RPE characteristics, especially with increasing passages, such as morphology, retinoid metabolism, and VEGF secretion. Furthermore, it should be mentioned that depigmentation in the RPE of AMD eyes is different from that of ARPE-19 cells. Melanosome density in the RPE decreases significantly with normal aging and more evidently in AMD, but melanin is not completely lost. More importantly, RPE melanin in AMD loses its antioxidant properties [31].

RPE cells in vivo form the outer blood-brain barrier and support photoreceptor cells and enable phototransduction. The outer blood-brain barrier is formed of a continuous layer of tight junctions that enable transepithelial transport and phototransduction. Pigment-Epithelium-Derived Factor (PEDF), which maintains barrier integrity and is endogenously secreted by RPE in vivo in large amounts, shows only basal levels in aged eyes. The in vitro ARPE-19 cell line is a rapidly growing immortalized human cell line derived from primary RPE cells from the globes of a 19-year-old male donor. ARPE-cell lines express RPE cell-specific markers CRALBP and RPE65 and form a viable cuboidal to columnar epithelium monolayer in culture media. Our recent study characterized and confirmed the expression of the following RPE-specific markers in the ARPE-19 cell line used in our lab: Cellular retinaldehyde binding protein-1 (CRALBP), Bestrophin1 (BEST1), and Keratin-18 (KRT18) [32]. Although ARPE-19 cells retain the characteristic features of RPE, including defined cell borders and pigmentation, they require considerable time for differentiation and are unable to completely differentiate into RPE-like layers found in vivo. ARPE-19 cells show partial polarization as some of the cells in the monolayer resemble the morphology of differentiated RPE cells such as apical microvilli, polarized distribution of organelles, basolateral infoldings, and junctional complexes on the apical plasma membrane. ARPE-19 cells exhibit low transepithelial resistance (TER) that reaches a maximum value of 50–100 Ω cm^2^ after 28 days of culture in low-serum media in laminin-coated Transwell-COL filters. It is speculated that the low TER might be due to heterogeneity of the cell line since some of the cells show polarization [33].

The present study demonstrated that treatment of ARPE-19 cells with anti-VEGF drugs altered the total HDAC protein activity and the gene expression levels of apoptotic, inflammatory, and oxidative stress-related genes. Moreover, addition of Trichostatin-A (TSA), an HDAC inhibitor, along with an anti-VEGF drug modulated the gene expression of *VEGF*, apoptotic and inflammatory markers. These results suggest that epigenetics modulation in ARPE-19 cells is strongly influenced by anti-VEGF drug treatment.

## 2. Materials and Methods

### 2.1. ARPE-19 Cell Culture

Human RPE cells (ARPE-19 cells, ATCC, Manassas, VA, USA) were cultured till confluent in 175 cm^2^-flasks containing DMEM/F-12 culture medium (Dulbecco’s Modification of Eagle’s Medium, Mediatech, Inc., Manassas, VA, USA), 10% dialyzed fetal bovine serum, antibiotics (penicillin G 100 U/mL, streptomycin sulfate 0.1 mg/mL, gentamicin 10 μg/mL, amphotericin B 2.5 μg/mL) and 17.5 mM glucose. All ARPE-19 cells were at passage 10 and cultured side-by-side under identical standard conditions of 37 °C in 5% CO_2_ and 95% relative humidity, in order to avoid any potential technical variability.

ARPE-19 cells are a spontaneously arising RPE cell line derived by Amy Aotaki-Keen from the normal eyes of a 19-year-old male who died from head trauma in a motor vehicle accident in 1986. The ARPE-19 cell line, established using the cuboidal basal cell layer cultured in specific culture media, expresses the RPE-specific markers cellular retinaldehyde binding protein and RPE-65.

In our study we utilized ARPE-19 cells at passage 10 in all our experiments to ensure the cells retained acceptable fidelity.

### 2.2. Drug Treatment of ARPE-19 Cell Cultures

ARPE-19 cells were plated in triplicate for 24 h in 6-well plates at a density of 500,000 cells per well, culture media were removed and replaced with the same media containing anti-VEGF drugs: aflibercept, ranibizumab or bevacizumab at 1× and 2× concentrations of the clinical intravitreal dose (12.5 μL/mL and 25 μL/mL, respectively). The clinical dose was calculated by assuming that the amount of each drug clinically used in intravitreal injections distributes equally throughout the 4 mL human vitreous. Untreated cells were used as control.

In order to further explore the potential relationship between anti-angiogenic treatment and the acetylation status of the target genes expression, a subset of ARPE-19 cells was treated with trichostatin A (TSA), an inhibitor of histone deacetylase (HDAC) activity, at 0.3 μM concentration, or a combination of 1X bevacizumab plus 0.3 μM TSA.

The control and anti-VEGF treated cultures were incubated for an additional 24 h, then RNA was isolated to be used for Quantitative Real-Time PCR (qRT-PCR) analyses.

Proteins extracted from cultures of untreated ARPE-19 cells, as well as cells treated with 1X bevacizumab, 0.3 μM TSA, and a combination of both drugs were analyzed for HDAC activity as described below.

### 2.3. RNA Extraction, Amplification of cDNA, and Quantitative Real-Time PCR (qRT-PCR) Analysis

ARPE-19 cells were pelleted for RNA isolation using a PureLink RNA Mini Kit (Ambion, Thermo Fisher Scientific, Waltham, MA, USA). For qRT-PCR analyses, 100 ng of individual RNA samples were reverse transcribed into complementary DNA (cDNA) using SuperScript VILO Master Mix (Thermo Fisher Scientific, Waltham, MA, USA).

We investigated transcription profiles of downstream genes known to play a role in AMD pathogenesis. RNA samples were isolated from cells that were (a) untreated; (b) treated with 1× or 2× concentrations of the three anti-VEGF drugs; (c) treated with 0.3μM TSA alone; and (d) treated with 1× bevacizumab plus TSA (bevacizumab/TSA). qRT-PCR was performed using primers for downstream pathway genes, including angiogenesis (*VEGF-A* and *HIF-1α*), apoptosis (*BCL2L13, CASPASE-3,* and *CASPASE-9*), inflammation (*IL-18* and *IL-1β*) and oxidative stress (*GPX3A* and *SOD2*) (Table 1). The qRT-PCR was performed on individual samples using QuantiFast SYBR Green PCR Kit (Qiagen, Inc., Germantown, MD, USA) on StepOne Plus Real-Time PCR system (Thermo Fisher Scientific, Waltham, MA, USA). For the various target genes, housekeeping genes that had comparable amplification efficiencies were chosen in order to maximize the accuracy of our ΔΔCT values. The housekeeper genes were either hypoxanthine phosphorbosyltransferase 1 (HPRT1) or hydroxymethylbilane synthase (HMBS). Untreated samples were used as control. ΔΔCts (differences in cycle thresholds) were obtained and folds calculated using the formula 2^ΔΔCt^.

### 2.4. Protein Extraction and HDAC Activity Assay

ARPE-19 cell samples were lysed using RIPA buffer (Cat. #89900, Life Technologies Corp., Calsbad, CA, USA), supernatants were transferred to a new microfuge tube and protein concentrations were measured using the Bio-Rad DC Protein Assay system (Bio-Rad Laboratories, Richmond, CA, USA) according to the manufacturer’s instructions. Protein samples were kept in a −80 °C freezer until time of use in the HDAC activity assay.

HDAC activity in the protein samples was measured using the Fluorometric Histone Deacetylase Assay Kit (Sigma–Aldrich, St. Louis, MO, USA) according to the manufacturer’s protocol. Briefly, protein samples, assay buffer and HDAC substrate solution were added to the wells of a 96-well plate. Each well contained 20 μL of the protein sample, 30 μL of assay buffer and 50 μL of HDAC substrate solution. The plate was incubated at 30 °C for 30 min, then 10 μL of Developer solution added to each well. The plate was incubated 10 min at room temperature, and fluorescence measured with a microplate spectrofluorometer (Gemini XPS, Molecular Devices, Sunnyvale, CA, USA) using an excitation wavelength of 350 nm and an emission wavelength of 440 nm. Samples were plated in triplicate and Hela cell lysate used as a positive control for HDAC activity. Experiments were performed on three replicates i.e., in triplicate. The plate in which the cells were plated was read three times and the fluorescence intensity was averaged. The entire experiment, i.e., treatment and reading the fluorescence was repeated three times to ensure reproducibility. Protein samples from untreated ARPE-19 cells were used as control and were normalized to 100%.

### 2.5. Statistical Analyses

Statistical analyses of the data were performed by unpaired t-test using GraphPad Prism, Version 5 (GraphPad Software, Inc., La Jolla, CA, USA). *p* < 0.05 was considered statistically significant. Untreated samples (controls) were normalized to a value of 100% for comparison to treated samples.

## 3. Results

### 3.1. Measurement of HDAC Activity in ARPE-19 Cells before and after Treatment with Anti-VEGF and TSA

**Figure 1:**
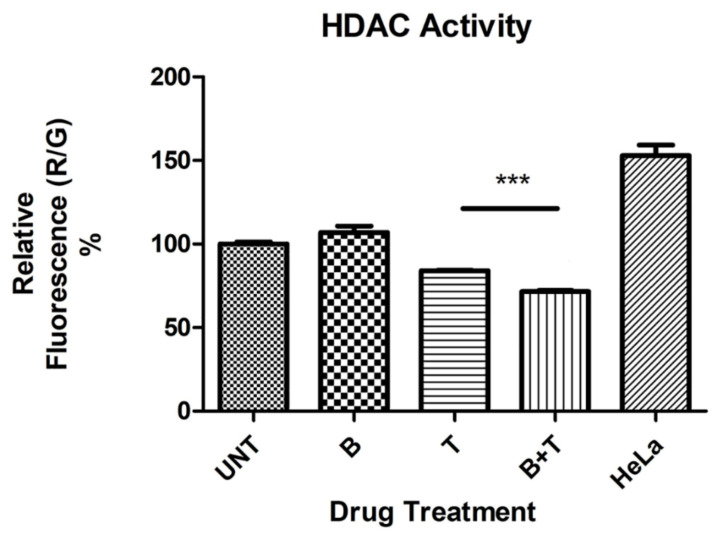
Histone deacetylase (HDAC) activity as determined by relative fluorescence (%) in untreated and treated ARPE-19 cultures. *** *p* < 0.001; Bars with no asterisk represent nonsignificant difference. UNT: Untreated; B: Bevacizumab 1× conc.; T: Trichostatin A 0.3 μM; B+T: Bevacizumab 1× conc. + Trichostatin A 0.3 μM. HeLa: Hela cell lysate positive control for HDAC activity. Error bars represent ‘Mean ± SEM’.

Treatment of ARPE-19 cells with 0.3 μM TSA resulted in significant reduction of HDAC activity (*p* = 0.0003), as did the combination of bevacizumab 1× plus TSA (*p* < 0.0001) (Figure 1). Although treatment with bevacizumab 1X alone did not significantly alter HDAC activity (*p* = 0.15), its addition to TSA significantly potentiated its inhibition of HDAC activity (*p* = 0.0001).

### 3.2. Effect of Anti-VEGF Treatment and HDAC Inhibition on the Expression Profiles of Downstream Genes

**Figure 2:**
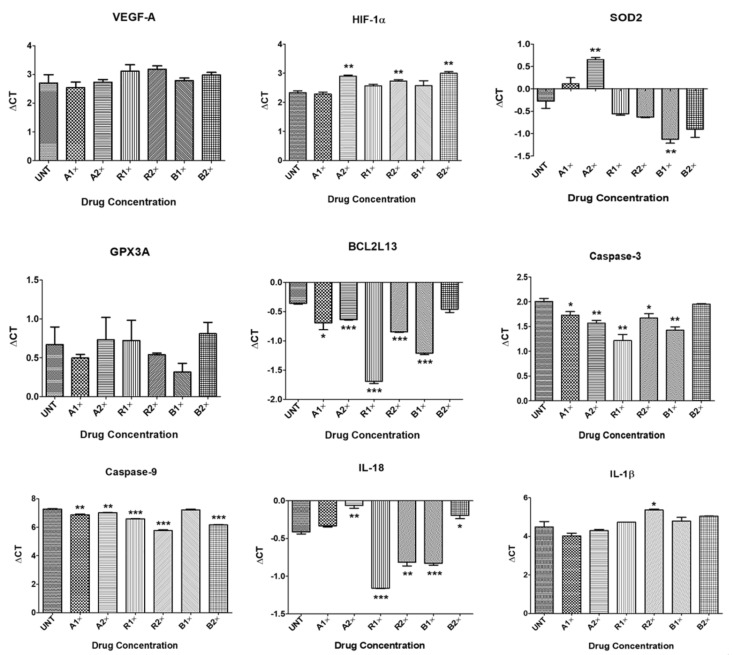
Quantitative Real-Time PCR (qPCR) data showing Delta Cts for downstream genes in untreated and anti-VEGF treated ARPE-19 cultures. * *p* < 0.05; ** *p* < 0.01; *** *p* < 0.001; Bars with no asterisk represent nonsignificant difference. UNT: Untreated; A1×: Aflibercept 1× conc.; A2×: Aflibercept 2× conc.; R1×: Ranibizumab 1× conc.; R2×: Ranibizumab 2× conc.; B1×: Bevacizumab 1× conc.; B2×: Bevacizumab 2× conc. Error bars represent ‘Mean ± SEM’.

Aflibercept 1×-treated ARPE-19 cells showed upregulation of *VEGF-A* (1.12-fold, *p* = 0.67), *HIF-1α* (1.03-fold, *p* = 0.63), *GPX3A* (1.13-fold, *p* = 0.49), *BCL2L13* (1.26-fold, *p* = 0.05), *CASPASE-3* (1.21-fold, *p* = 0.04), *CASPASE-9* (1.32-fold, *p* = 0.003) and *IL-1β* (1.38-fold, *p* = 0.22); and downregulation of *SOD2* (0.76-fold, *p* = 0.14) and *IL-18* (0.95-fold, *p* = 0.051), compared to untreated cells (Table 2, Figure 2).

Treatment with aflibercept 2× downregulated the expression of *VEGF-A* (0.98-fold, *p* = 0.94), *HIF-1α* (0.67-fold, *p* = 0.002), *SOD2* (0.52-fold, *p* = 0.005), *GPX3A* (0.96-fold, *p* = 0.87), and *IL-18* (0.78-fold, *p* = 0.002), and led to upregulation of *BCL2L13* (1.21-fold, *p* < 0.0001), *CASPASE-3* (1.36-fold, *p* = 0.006), *CASPASE-9* (1.19-fold, *p* = 0.004) and *IL-1β* (1.14-fold, *p* = 0.55) (Table 2, Figure 2).

ARPE-19 cells treated with ranibizumab 1× showed downregulation of *VEGF-A* (0.75-fold, *p* = 0.40), *HIF-1α* (0.85-fold, *p* = 0.05), *GPX3A* (0.96-fold, *p* = 0.89), and *IL-1β* (0.84-fold, *p* = 0.43) and upregulation of *SOD2* (1.22-fold, *p* = 0.16), *BCL2L13* (1.21-fold, *p* > 0.0001), *CASPASE-3* (1.73-fold, *p* = 0.004), *CASPASE-9* (1.61-fold, *p* < 0.0001) and *IL-18* (1.67-fold, *p* < 0.0001) (Table 2, Figure 2).

Ranibizumab 2×-treated cells showed downregulation of *VEGF-A* (0.72-fold, *p* = 0.20), *HIF-1α* (0.76-fold, *p* = 0.008) and *IL-1β* (0.54-fold, *p* = 0.04), and upregulation of *SOD2* (1.28-fold, *p* = 0.09), *GPX3A* (1.09-fold, *p* = 0.60), *BCL2L13* (1.4-fold, *p* < 0.0001), *CASPASE-3* (1.26-fold, *p* = 0.04), *CASPASE-9* (2.82-fold, *p* < 0.0001) and *IL-18* (1.32-fold, *p* = 0.002) (Table 2, Figure 2).

Treatment with bevacizumab 1× decreased the expression of *VEGF-A* (0.95-fold, *p* = 0.80), *HIF-1α* (0.85-fold, *p* = 0.26) and *IL-1β* (0.81-fold, *p* = 0.42), and increased the expression of *SOD2* (1.8-fold, *p* = 0.009), *GPX3A* (1.28-fold, *p* = 0.23), *BCL2L13* (1.8-fold, *p* < 0.0001), *CASPASE-3* (1.5-fold, *p* = 0.003), *CASPASE-9* (1.04-fold, *p* = 0.52) and *IL-18* (1.33-fold, *p* = 0.0003) (Table 2, Figure 2).

Bevacizumab 2× resulted in downregulation of *VEGF-A* (0.82-fold, *p* = 0.41), *HIF-1α* (0.63-fold, *p* = 0.001), *GPX3A* (0.91-fold, *p* = 0.63), *IL-18* (0.86-fold, *p* = 0.02), and *IL-1β* (0.68-fold, *p* = 0.12); and upregulation of *SOD2* (1.54-fold, *p* = 0.06), *BCL2L13* (1.07-fold, *p* = 0.16), *CASPASE-3* (1.04-fold, *p* = 0.39), and *CASPASE-9* (2.16-fold, *p* < 0.0001) (Table 2, Figure 2).

**Figure 3:**
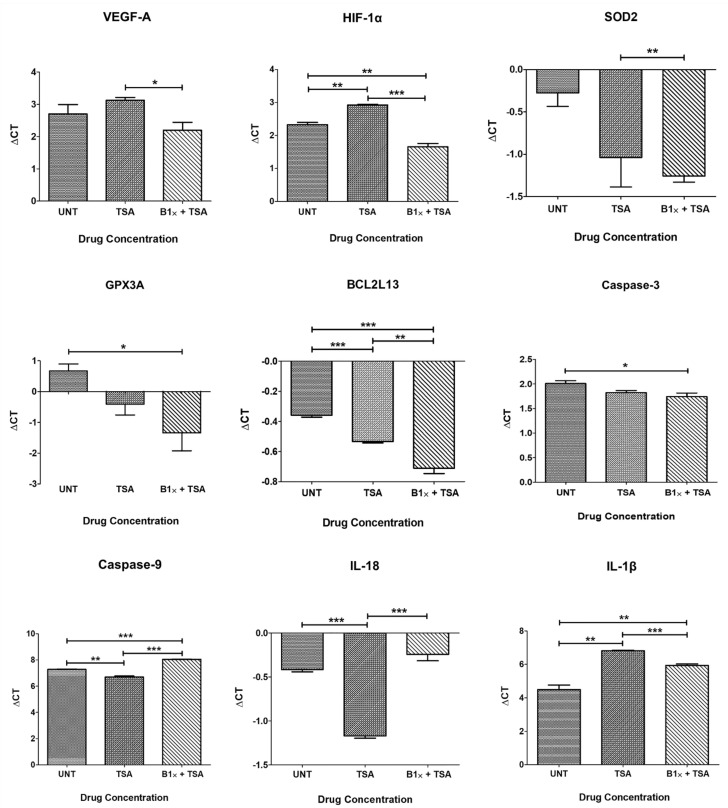
Quantitative Real-Time PCR (qPCR) data showing Delta Cts for downstream genes after treatment with Trichostatin A and Bevacizumab. * *p* < 0.05; ** *p* < 0.01; *** *p* < 0.001; Bars with no asterisk represent nonsignificant difference. UNT: Untreated; TSA: Trichostatin A 0.3 μM; B1×+TSA: Bevacizumab 1× conc. + Trichostatin A 0.3 μM. Error bars represent ‘Mean ± SEM’.

TSA treated (0.3 μM) ARPE-19 cultures resulted in downregulation of *VEGF-A* (0.75-fold, *p* = 0.24), *HIF-1α* (0.66-fold, *p* = 0.001) and *IL-1β* (0.2-fold, *p* = 0.001), and caused upregulation of *SOD2* (1.69-fold, *p* = 0.11), *GPX3A* (2.11-fold, *p* = 0.06), *BCL2L13* (1.13-fold, *p* = 0.0003), *CASPASE-3* (1.14-fold, *p* = 0.07), *CASPASE-9* (1.49-fold, *p* = 0.003) and *IL-18* (1.69-fold, *p* < 0.0001) compared to untreated cells (Table 3, Figure 3).

The combination of bevacizumab 1× plus TSA resulted in upregulation of *VEGF-A* (1.42-fold, *p* = 0.25), *HIF-1α* (1.60-fold, *p* = 0.006) *SOD2* (1.97-fold, *p* = 0.005), *GPX3A* (4.03-fold, *p* = 0.03), *BCL2L13* (1.28-fold, *p* = 0.0007) and *CASPASE-3* (1.2-fold, *p* = 0.05), and decreased expression of *CASPASE-9* (0.59-fold, *p* = 0.09), *IL-18* (0.89-fold, *p* = 0.09) and *IL-1β* (0.37-fold, *p* = 0.008) compared to untreated cells (Table 3, Figure 3).

When comparing cells treated with both drugs to cells treated with TSA alone, the addition of bevacizumab 1× significantly reversed the effect of TSA on the expression of *VEGF-A* (*p* = 0.02), *HIF-1α* (*p* = 0.0003), *CASPASE-9* (*p* < 0.0001), and *IL-18* (*p* = 0.0003), reduced its effect on an *IL-1β* (*p* = 0.0006), and potentiated its effect on *BCL2L13* (*p* = 0.008) (Figure 3).

## 4. Discussion

In this study, we demonstrated differential HDAC total protein activity in response to anti-VEGF drug treatment in ARPE-19 cells. Treatment of ARPE-19 cells with anti-VEGF drugs alone in varying concentrations as well as additive effects of TSA and anti-VEGF drug significantly modulated the gene expression profiles of apoptotic, inflammatory, and oxidative stress markers. Therefore, our results suggest that addition of anti-VEGF drugs to cultured ARPE-19 cells strongly influence the regulation of epigenetic markers and downstream molecular markers.

Environmental stimuli are thought to induce epigenetic changes that accumulate in the cell with increasing age, as evidenced by age being the major risk factor for AMD, as well as the discordance of disease phenotype in identical twins that possess similar risk profiles for AMD [34]. Hypermethylation of genes encoding for reactive oxygen species (ROS) scavengers, namely *GSTM1* and *GSTM5*, was demonstrated in AMD RPE and choroid, downregulating their expression and rendering the cells more susceptible to oxidative damage. Additionally, several micro RNAs (miRNAs) have been implicated in AMD pathogenesis through their contribution to aberrant angiogenesis, inflammation, and apoptosis in response to oxidative stress both in vitro and in vivo [22]. Hypomethylation of the promoter region of interleukin 17 receptor C (*IL17RC*), a pro-inflammatory gene, promotes its expression and increases inflammation in AMD patients [35]. *Oliver* et al. conducted the first genome-wide epigenetic study in AMD and found hypomethylation at *HTRA/ARMS2* locus, which is a major susceptibility locus for AMD. They also observed hypermethylation of the *PRSS50* locus which had not been previously associated with AMD [23].

Over the last decade, intravitreal injection of anti-angiogenic drugs has been the mainstay of therapy for neovascular AMD as well as for macular edema associated with diabetic retinopathy and retinal vein occlusion [36]. The three drugs commonly used in clinical practice are bevacizumab, ranibizumab and aflibercept. Bevacizumab is a full-length monoclonal antibody that has a molecular weight of 149 kilodalton (kDa) and binds all isoforms of VEGF-A rendering them inactive. Ranibizumab is an antigen-binding Fab fragment of the same parent antibody as bevacizumab. It lacks the Fc domain and has a molecular weight of 48 kDa. Aflibercept, a 96.9 kDa recombinant fusion protein, contains immunoglobulin fragments from both VEGF receptors: VEGFR1 and 2, combined with an Fc antibody fragment. It acts as a decoy receptor that not only binds VEGF-A, but also, unlike the former two drugs, can bind VEGF-B and placental-like growth factor (PlGF) [37,38,39]. The different molecular weights and structures of the three drugs may have an effect on their ocular and systemic pharmacokinetics and pharmacodynamics [40]. All three drugs have been shown to accumulate intracellularly. Uptake of bevacizumab and aflibercept is mediated through their Fc portion, but ranibizumab is likely internalized through a VEGF/VEGFR2-mediated mechanism [30].

RPE dysfunction is central to the pathogenesis of AMD, and RPE cells are the main source of VEGF in the retina [41]. Many investigators have tested the safety of anti-VEGF agents on RPE cells both in vitro and in animal models. Our group previously demonstrated a good safety profile of the three anti-angiogenic drugs on ARPE-19 cell cultures at the clinical dose, but mild cytotoxic effects were found at higher doses [42,43]. Other studies have shown similar results [44,45].

A study by Dinc et al. in which APRE-19 cells were subjected to H_2_O_2_-induced oxidative stress and levels of miRNA expression were evaluated, suggested an epigenetic role for anti-VEGF drugs [46]. In that study, several miRNAs were dysregulated in response to oxidative stress compared to untreated samples. Preincubation of cells with any of the three anti-VEGF drugs before H_2_O_2_ treatment significantly altered the miRNA dysregulation induced by H_2_O_2_. The authors suggested that anti-VEGF drugs could protect RPE cells from oxidative stress through their effect on miRNAs.

We tried to determine whether the changes in *HDAC* expression would affect the activity of HDAC enzymes in ARPE-19 cells using an assay kit designed to measure the collective activity of all HDACs. Bevacizumab treatment alone failed to alter HDAC activity to a significant extent compared to untreated cultures. Our inability to observe a significant net effect on HDAC enzyme activity may be because bevacizumab had opposite effects on the expression of both *HDAC1* and *HDAC6* genes, so the overall HDAC activity after treatment was neutral. As expected, Trichostatin A (TSA) significantly inhibited HDAC activity. TSA is known to inhibit both class I and II HDACs. Interestingly, the combination of TSA plus bevacizumab increased significantly the frank inhibitory effect on HDAC activity, but the mechanisms for additive effects are not clear and need to be further investigated.

Next, the expression levels for genes known to regulate important pathogenetic pathways for AMD after treatment with anti-VEGF drugs and TSA were measured.

### 4.1. Angiogenesis Genes

HIF-1α is the oxygen-sensitive subunit of HIF-1, a transcription factor upregulated in cells in response to hypoxia. Activation of HIF-1α results in overexpression of downstream genes, including pro-angiogenic genes, mainly VEGF-A [47]. Both class I and II HDACs are upregulated by hypoxia and induce angiogenesis, particularly HDAC1 [48,49]. HIF-1α is a non-histone protein target for acetylation. It is acetylated under normoxic conditions, which decreases its stability by facilitating its proteasomal degradation. Under hypoxic conditions, HIF-1α becomes deacetylated by HDACs, which makes it more stable and prolongs its half-life [50].

We found that all three anti-VEGF drugs downregulated the expression of *HIF1-α* gene at 2×, but not 1×, concentration. This suggests that these drugs might protect cells against oxidative damage. Treatment with TSA had the same inhibitory effect. This observation comes in agreement with previous studies demonstrating that TSA could downregulate both gene and protein expression of HIF-1α in vitro and in animal models [51,52]. Other HDAC inhibitors demonstrated a protective effect against oxidative damage in vitro and animal models. These include valproic acid (VPA), which is another broad-spectrum HDACi, and tubastatin A (TST); a specific inhibitor of HDAC6 [53,54]. The inhibitory effect displayed by anti-VEGF drugs and TSA in our study was reversed by the combination of bevacizumab and TSA, which significantly upregulated *HIF1-α* expression.

VEGF-A is the main pro-angiogenic factor implicated in the pathogenesis of wet AMD [55]. The expression levels of VEGF, as well as tissue response to its secretion, are modulated by both genetic and epigenetic factors. Epigenetic regulation of angiogenesis has been extensively studied in cancer cells. Both HDAC7 and SIRT1 promote angiogenesis during development and disease by inducing pro-angiogenic factors and suppressing anti-angiogenic ones [56,57,58]. HDAC6 has also been associated with angiogenesis due to its ability to bind several cytoskeletal proteins in the cytoplasm and stimulate vascular proliferation and sprouting. The anti-angiogenic properties of HDAC inhibitors are the basis for their use as anti-cancer agents and extend beyond gene silencing to directly acetylating angiogenic factors in the cytoplasm. Chan and colleagues demonstrated that TSA could downregulate *VEGF-A* by epigenetically silencing its expression in the presence of CoCl_2_, a hypoxia-inducing agent, in human RPE cells in vitro. Furthermore, they showed that TSA could attenuate a laser induced CNV in a mouse model. However, we found that TSA did not alter *VEGF-A* gene expression in ARPE-19 cells. The ability of TSA to silence gene expression might be more pronounced under conditions of hypoxic stress that induce aberrant upregulation of VEGF-A, which was not the case in our experiment.

Two studies explored the effect of either ranibizumab or bevacizumab on *VEGF-A* gene expression in primary RPE cells. In one experiment, bevacizumab had no effect on the baseline expression levels of *VEGF-A* [59]. Similarly, in another study ranibizumab treatment did not significantly alter *VEGF-A* mRNA overexpression induced by white light illumination of RPE cells [60]. In ARPE-19 cells, both aflibercept and ranibizumab induced *VEGF-A* mRNA expression after 24 h of treatment. The authors postulated that the cells upregulated the transcription of *VEGF-A* to compensate for blocking the VEGF-A protein in the culture media [43]. Similarly, another study demonstrated that ranibizumab and bevacizumab induced overexpression of *VEGF-A* inARPE-19 cells that were subjected to oxidative stress by preincubation in a hypoxic chamber [61]. Another study showed a similar compensatory upregulation in response to bevacizumab treatment in murine RPE cells in vivo, but not in ARPE-19 cells in vitro. These investigators suggested that the compensatory upregulation of *VEGF-A* expression in complex in vivo systems might not be captured by the simpler APRE-19 cell model. It could also be that the RPE of healthy young animals was able to compensate for VEGF-A neutralization, unlike ARPE-19 cells, which carry more similarities to aged RPE [62]. Other studies showed that anti-VEGF drugs could bring down *VEGF-A* gene expression to control levels in human retinal pericytes [63] and in ARPE-19 cells [19].

*VEGF-A* expression levels were not affected by any of the used drugs in our experiment, although its expression was significantly lower in cells treated with a combination of bevacizumab plus TSA compared to treatment with TSA alone. It seems that the effect of anti-angiogenic drugs on *VEGF-A* gene expression varies according to cell type, the nature of biologic stress that the cells are exposed to as well as the in vivo versus in vitro environment.

### 4.2. Oxidative Stress Genes

Oxidative stress occurs when ROS accumulation overwhelms the capacity of the cell to detoxify them. Neural and RPE cells in the retina have a high metabolic demand and are most prone to oxidative damage with aging. Aging is associated with differential gene expression and chromatin reorganization mediated by epigenetic mechanisms, ultimately leading to impaired ability of the cells to adapt to environmental stress [64].

The main antioxidant in the retina is the superoxide dismutase (SOD) family [65]. *SOD2* gene encodes for a mitochondrial enzyme, which deactivates superoxide free radicals. Polymorphisms of *SOD2* have been associated with exudative AMD [66]. SOD2 expression depends on acetylation. Being a mitochondrial enzyme, SOD2 is directly deacetylated by SIRT3, a mitochondrial Class III HDAC, resulting in its activation [67]. Forkhead box O3a (FoxO3a) activates the promoter of *SOD2* gene inducing its expression. TSA was shown to increase the acetylation of *FoxO3a* promoter region, and upregulating its expression, as well as its target protein, SOD2, expression in vitro [68]. Treatment with bevacizumab 1× in our study significantly upregulated *SOD2* expression, as did combined treatment with bevacizumab and TSA. Conversely, aflibercept significantly reduced *SOD2* expression.

Glutathione peroxidase (GPX) is another antioxidant enzyme found in the RPE and photoreceptors that protects the retina from oxidative damage. GPX expression is upregulated in AMD patients, most likely due to oxidative stress [69]. VPA induced the expression of *SOD2* and *GPX* genes in ARPE-19 cells in normal conditions and maintained their expression hypoxic conditions. A similar effect was demonstrated in rat retina [70]. In our study, only the combination of bevacizumab plus TSA was able to significantly increase *GPX3A* expression.

### 4.3. Inflammation Genes

Inflammation has been recently recognized as a key player in the pathogenesis of AMD. Both drusen components and intracellular lipofuscin can incite inflammasome activation and the release of the pro-inflammatory cytokines IL-18 and IL-1β in retinal tissues [71,72]. It is thought that epigenetic mechanisms are involved in initiating the immune response by altering the gene expression of immune cells to allow for cytokine production and chemotaxis [24]. HDAC6 inhibition by TST suppressed mRNA expression of *IL-1β* in an inflammation model of mammary epithelial cells in vitro [73]. We found that TSA treatment had the same effect on *IL-1β* gene expression in ARPE-19 cells. Cultures treated with ranibizumab 2× and the TSA+bevacizumab combination also displayed downregulation of *IL-1β*.

Although inflammation significantly contributes to tissue damage in AMD, a certain degree of inflammation might be needed to protect against neovascularization. IL-18 has exhibited anti-angiogenic properties in tumors and post-ischemic injury and is being investigated as a potential anti-angiogenic therapy in wet AMD [74]. Shen et al. demonstrated reciprocal suppression between VEGF and IL-18. They were able to detect increased levels of IL-18 in the aqueous of patients receiving intravitreal ranibizumab injections for macular edema secondary to retinal vein occlusion. Furthermore, they found that intravitreal injection of anti-VEGF antibody in a mouse model of ischemic retinopathy upregulated mRNA expression of *IL-18*. IL-18 was able to block VEGF-induced vascular leakage and neovascularization in mice. Thus, each agent can suppress both the production and function of the other [75]. This observation could explain our findings that *IL-18* was significantly upregulated by both concentrations of ranibizumab and bevacizumab. TSA also upregulated *IL-18* expression, possibly owing to its anti-angiogenic properties. Aflibercept 2×, however, suppressed its expression. Aflibercept has a different molecular structure than both ranibizumab and bevacizumab and could have triggered another signaling mechanism that reduced *IL-18* transcription.

### 4.4. Apoptosis Genes

RPE cell loss characteristic of advanced AMD is thought to represent cell death by apoptosis. The BCL-2 family regulates the intrinsic mitochondrial pathway of apoptosis. Accumulation of oxidized low-density lipoproteins (LDL) in drusen and basal linear deposits, both hallmark lesions of AMD, results in upregulation of BAX, a pro-apoptotic BCL-2 family member, and downregulation of BCL-2, an anti-apoptotic member. The increased BAX/BCL-2 ratio tips the balance in favor of apoptosis [76]. Activation of pro-apoptotic BCL-2 members results in opening of mitochondrial membrane pores with subsequent release of pro-apoptotic factors, such as cytochrome c, into the cytoplasm. Cytochrome c can recruit and activate caspase-9, an initiator caspase, which activates effector caspases, such as caspase-3, that eventually cause degradation of genomic DNA and cell death [77].

Anti-VEGF drug treatment of ARPE-19 cells resulted in upregulation of the 4 pro-apoptotic genes in this study. This effect was seen with the three drugs tested at both 1× and 2× concentrations. We previously demonstrated some degree of reduced mitochondrial membrane potential (MMP), which is an early sign of apoptosis, in ARPE-19 cells after 24 h of treatment with higher-than-clinical concentrations of ranibizumab and aflibercept. Only bevacizumab decreased MMP at 1× concentration [42]. Another study found that bevacizumab significantly increased apoptosis in an ARPE-19 cell model of oxidative stress and as stress levels increased, the dose of bevacizumab capable of inducing apoptosis decreased. The authors postulated that bevacizumab blocked the protective effects of VEGF under high oxidative stress conditions and downregulated *BCL-2* gene expression [78]. Another study showed that ranibizumab could enhance the anti-proliferation effects of oxidative stress on ARPE-19 cells [42].

These results warrant further in vivo investigations since the net effect on retinal cells in vivo is subject to a complex interplay of many protective and detrimental factors. Anti-angiogenic therapy has demonstrated protective effects in our in vitro study, as evidenced by suppressing oxidative stress and inflammatory cytokine gene transcription. However, further research is warranted as concerns have been raised about the development of geographic atrophy in 98% of wet AMD patients receiving chronic anti-VEGF injections over prolonged periods of time [79].

## 5. Conclusions

In conclusion, we demonstrated that anti-VEGF drugs can (1) alter expression profiles for genes involved in oxidative stress, inflammation, and apoptosis pathways and (2) modulate intracellular signal transduction in addition to blocking VEGF-A. This could have implications in management of resistance or nonresponse to anti-VEGF therapy in some AMD patients. The phenomenon of individual variation in response to anti-VEGF treatment has also been observed in different cancers treated with bevacizumab [80,81,82]. Genetic variations may render vascular tissue more responsive or resistant to drug effects. Epigenetic mechanisms may render tissues less sensitive to the anti-VEGF treatment and influence pharmacogenetic interactions, as evidenced by miRNA regulation of enzymes involves in drug uptake and metabolism [83,84]. The fact that these drugs could influence the epigenome might guide precision medicine in the future by obtaining an “epigenetic” profile for wet AMD patients to predict resistance and direct the choice of therapy.

Another avenue we believe is worth exploring is the possibility of adding HDAC inhibitors to the therapeutic armamentarium of AMD. Epigenetic drugs have shown a great promise in immunomodulation, neuroprotection, and angiogenesis suppression [85]. To date, 6 epigenetic drugs have been approved by the FDA for cancer therapy [51]. A variety of epigenetic drugs, including DNMT and HDAC inhibitors are currently under investigation as potential therapeutic agents in AMD, owing to their ability to reverse inflammation and angiogenesis [55,86]. Specific HDAC inhibitors might be preferable to pan-inhibitors, such as TSA, as the latter can cause undesirable alterations in gene expression by inducing histone hyperacetylation [53].

Further studies including in vivo tests are required. Other retinal cell types are involved in the evolution of AMD pathogenesis, and the impact of the studied drugs on these cells needs to be explored as well. Human pluripotent stem cell (hPSC)-derived retinal organoids could provide an alternative platform to study drug interactions and intracellular signaling mechanisms that more closely approximates the retinal environment in vivo [87].

## Figures and Tables

**Table 1:** Gene symbols, names, gene bank accession numbers, and functions.

Gene Symbol ^a^	Gene Name ^b^	Gene Bank Accession Num. ^c^	Function ^d^
***VEGF-A***	Vascular endothelial growth factor A	NM_001025366, NM_001025367, NM_001025368, NM_001033756, NM_001171623, NM_001171624, NM_001171625, NM_001171626, NM_001171629, NM_003376, NM_001287044	Member of PDGF/VEGF growth factor family and encodes a protein that specifically acts on endothelial cells, mediating increased vascular permeability, inducing angiogenesis, vasculogenesis, and endothelial cell growth, promoting cell migration, and inhibiting apoptosis.
***HIF-1α***	Hypoxia inducible factor 1 alpha	NM_001243084, NM_001530, NM_181054	Master regulator of cellular and systemic homeostatic response to hypoxia by activating transcription of many genes, including those involved in energy metabolism, angiogenesis, apoptosis, and other genes whose protein products increase oxygen delivery or facilitate metabolic adaptation to hypoxia.
***SOD2***	Superoxide dismutase 2, mitochondria	NM_000636 NM_001024465	Binds to the superoxide byproducts of oxidative phosphorylation and converts them to H_2_O_2_ and O_2_.
***GPX3***	Glutathione peroxidase 3	NM_002084	Catalyzes the reduction of hydrogen peroxide.
***BCL2L13***	BCL2-like 13 (apoptosis facilitator)	NM_015367	Encodes a mitochondrially-localized protein with conserved B-cell lymphoma 2 homology motifs. Overexpression of the encoded protein results in apoptosis.
***CASPASE-3***	Caspase 3	NM_004346, NM_032991	The protein encoded by this gene is a cysteine-aspartic acid protease that plays a central role in the execution phase of cell apoptosis. It cleaves and inactivates poly (ADP-ribose) polymerase while it cleaves and activates sterol regulatory element binding proteins, as well as caspases 6, 7, and 9. This protein itself is processed by caspases 8, 9, and 10. It is the predominant caspase involved in the cleavage of amyloid-beta 4A precursor protein, which is associated with neuronal death in Alzheimer’s disease
***CASPASE-9***	Caspase 9	NM_001229, NM_032996	This gene encodes a member of the cysteine-aspartic acid protease (caspase) family that plays a central role in the execution phase of cell apoptosis. This protein can undergo autoproteolytic processing and activation by the apoptosome, a protein complex of cytochrome c and the apoptotic peptidase activating factor 1; this step is thought to be one of the earliest in the caspase activation cascade. This protein is thought to play a central role in apoptosis and to be a tumor suppressor.
***IL-18***	Interleukin 18	NM_001243211 NM_001562	Proinflammatory cytokine that augments natural killer cell activity in spleen cells and stimulates interferon gamma production in T-helper type I cells.
***IL-1β***	Interleukin 1, beta (also known as IL-1β)	NM_000576, XM_006712496	Produced by activated macrophages as a proprotein and processed to its active form by caspase 1 (CASP1/ICE). It is an important mediator of the inflammatory response; and is involved in cell proliferation, differentiation, and apoptosis

**^a^** Official gene symbol by HUGO (Human Genome Organization) Gene Nomenclature Committee (HGNC). **^b^** Official gene name by HUGO Gene Nomenclature Committee (HGNC). **^c^** Gene Accession Bank Number from the primers used (Qiagen, Valencia, CA). **^d^** Gene function modified from PubMed gene.

**Table 2:** Expression folds for downstream genes in untreated and anti-vascular endothelial growth factor (anti-VEGF)-treated ARPE-19 cultures *.

	Aflibercept				Ranibizumab				Bevacizumab			
	1×		2×		1×		2×		1×		2×	
Gene	Fold	*p*-value	Fold	*p*-value	Fold	*p*-value	Fold	*p*-value	Fold	*p*-value	Fold	*p*-value
**Angiogenesis**												
*VEGF-A*	1.12	0.67	0.98	0.94	0.75	0.40	0.72	0.20	0.95	0.80	0.82	0.41
*HIF-1α*	1.03	0.63	0.67	*0.002*	0.85	0.05	0.76	*0.008*	0.85	0.26	0.63	*0.001*
**Antioxidant**												
*SOD2*	0.76	0.14	0.52	*0.005*	1.22	0.16	1.28	0.09	1.80	*0.009*	1.54	0.06
*GPX3A*	1.13	0.49	0.96	0.87	0.96	0.89	1.09	0.60	1.28	0.23	0.91	0.63
**Apoptosis**												
*BCL2L13*	1.26	*0.05*	1.21	*<0.0001*	1.21	*<0.0001*	1.40	*<0.0001*	1.80	*<0.0001*	1.07	0.16
*CASPASE-3*	1.21	*0.04*	1.36	*0.006*	1.73	*0.004*	1.26	*0.04*	1.50	*0.003*	1.04	0.39
*CASPASE-9*	1.32	*0.003*	1.19	*0.004*	1.61	*<0.0001*	2.82	*<0.0001*	1.04	0.52	2.16	*<0.0001*
**Inflammation**												
*IL-18*	0.95	0.051	0.78	*0.002*	1.67	*<0.0001*	1.32	*0.002*	1.33	*0.0003*	0.86	*0.02*
*IL-1β*	1.38	0.22	1.14	0.55	0.84	0.43	0.54	*0.04*	0.81	0.42	0.68	0.12

* Fold change was calculated using the formula: 2^ΔΔCT^. Untreated samples had a value of 1.

**Table 3:** Expression folds of downstream genes after treatment with Trichostatin A alone and in combination with Bevacizumab 1× *.

	TSA Compared to Untreated		Bevacizumab 1×+TSA Compared to Untreated	
Gene	Fold	*p*-value	Fold	*p*-value
**Angiogenesis**				
*VEGF-A*	0.75	0.24	1.42	0.25
*HIF-1α*	0.66	*0.001*	1.60	*0.006*
**Antioxidant**				
*SOD2*	1.69	0.11	1.97	*0.005*
*GPX3A*	2.11	0.06	4.03	*0.03*
**Apoptosis**				
*BCL2L13*	1.13	*0.0003*	1.28	*0.0007*
*CASPASE-3*	1.14	0.07	1.20	*0.05*
*CASPASE-9*	1.49	*0.003*	0.59	*<0.0001*
**Inflammation**				
*IL18*	1.69	*<0.0001*	0.89	0.09
*IL-1β*	0.20	*0.001*	0.37	*0.008*

* Fold change was calculated using the formula: 2^ΔΔCT^. Untreated samples had a value of 1.

## Data Availability

All data are presented within the manuscript.
